# Steering by their own lights: Why regulators across Europe use different indicators to measure healthcare quality

**DOI:** 10.1016/j.healthpol.2020.02.012

**Published:** 2020-05

**Authors:** Anne-Laure Beaussier, David Demeritt, Alex Griffiths, Henry Rothstein

**Affiliations:** a Centre de Sociologie des Organisations (CSO), Sciences Po-CNRS, 19 Rue Amélie, 75007 Paris, France; b Department of Geography, King’s College London, Strand, London WC2R 2LS, United Kingdom; c Data Science Directorate, Statica Research, London, SE22 9PN, United Kingdom

**Keywords:** Healthcare quality governance and improvement, Hospital quality indicators, Performance measurement, Comparative health policy

## Abstract

Despite widespread faith that quality indicators are key to healthcare improvement and regulation, surprisingly little is known about what is actually measured in different countries, nor how, nor why. To address that gap, this article compares the official indicator sets--comprising some 1100 quality measures-- used by statutory hospital regulators in England, Germany, France, and the Netherlands. The findings demonstrate that those countries’ regulators strike very different balances in: the dimensions of quality they assess (e.g. between safety, effectiveness, and patient-centredness); the hospital activities they target (e.g. between clinical and non-clinical activities and management); and the ‘Donabedian’ measurement style of their indicators (between structure, process and outcome indicators). We argue that these contrasts reflect: i) how the distinctive problems facing each country’s healthcare system create different ‘demand-side’ pressures on what national indicator sets measure; and ii) how the configuration of national healthcare systems and governance traditions create ‘supply-side’ constraints on the kinds of data that regulators can use for indicator construction. Our analysis suggests fundamental differences in the meaning of quality and its measurement across countries that are likely to impede international efforts to benchmark quality and identify best practice.

## Highlights

• Indicator sets differ in how they define, measure, and assess healthcare quality.
• National sets shaped by varying governance traditions and healthcare system configuration.
• Targeting of quality dimensions and hospital activities shaped by system-specific ‘demand-side’ pressures.
• Measurement styles shaped by ‘supply-side’ constraints on data access and indicator construction.
• International benchmarking is easier when healthcare systems and governance traditions are similar.

## 1 Introduction

Lord Kelvin [1] famously opined that only “when you can measure what you are speaking about and express it in numbers” can you “know something about it”. No doubt he would have applauded how healthcare quality, long regarded as too ineffable to define [2], is now subject to pervasive measurement to support everything from quality assurance and improvement to patient choice and payment by results. Indeed, quantitative indicators are now central to an international ‘quality movement’ [[3], [4], [5], [6], [7]], which has emerged over the last 25 years in response to spiralling costs, safety scandals and demands for more responsive and accessible care. In England’s National Health Service (NHS) for example, the number of performance indicators has skyrocketed from 70 in 1982 to more than 2000 today [8,9]. Likewise in the US the number of healthcare quality indicators endorsed by the National Quality Forum has more than doubled over the last decade to 1078 [10,11].

Measurement may be the first step to improvement [12], but the proliferation of indicators creates its own problems. For one thing, measurement and reporting are costly [10], with one recent study estimating the burden on a major US medical centre at 1% of total revenue [13], despite efforts to rationalise and reduce excessive reporting [4,14]. In turn, there are many competing ways of conceptualizing and measuring quality and of selecting, normalising, aggregating, and visualising quality indicators [[15], [16], [17], [18], [19]]. This creates difficulties in benchmarking performance-- both of individual providers [[20], [21], [22], [23]] and entire healthcare systems [[24], [25], [26]] —and can lead to poor choices by patients, policymakers, and practitioners alike [27].

To improve the consistency, reliability, and validity of care quality metrics, the World Health Organization, European Commission, OECD, and Institute of Medicine have published alternative frameworks for defining key indicator sets [10,[28], [29], [30]]. Indeed, the European Commission regards the development of comparable national indicator sets as vital to helping patients exercise their rights to accessing cross-border healthcare (2011/24/EU). However, despite efforts to create universal indicators that could help facilitate convergence and discourage countries from steering by their own lights [31,32], high-level international comparative studies have tentatively pointed to considerable unevenness in the selection and use of indicators across countries [7].

Part of the problem is that international frameworks tend to regard care quality as an objective phenomenon for which universally applicable measures can—and should-- be adopted regardless of the institutional contexts and purposes for which it is being assessed. Yet, as Pollitt et al. [8] have observed in relation to the general measurement of healthcare system performance, patterns of indicator adoption and use can depend on the distinctive problems facing different countries’ healthcare systems and governance traditions. For example, Pollitt et al. [8] suggest that governments within pluralist political systems face fewer institutional constraints than governments in corporatist political systems built on compromise among the social partners. Certainly, a number of high-level cross-national comparisons have pointed to distinctive national variation in the philosophies and regulatory mandates that underpin indicator use as well as the sources on which indicator sets draw and the purposes to which they are put [[33], [34], [35], [36]].

Beyond high-level observation, however, little is known about international variation in what actually gets measured and how, whether there are any distinctive patterns to that variation, and what might explain such variation. In order to address those lacunae, we undertake the first indicator-by-indicator comparison of the official sets used across advanced healthcare systems, examining four neighbouring EU countries: England, Germany, France and the Netherlands. We focus on the statutory regulation of acute hospital care because that is the area of healthcare provision where international efforts to define and measure quality are most advanced. In so doing, we consider whether and how the availability, design, and selection of quality measures vary and what those patterns reveal about regulatory priorities, institutional barriers to quality monitoring, and fundamental understandings of quality itself. We conclude by reflecting on the opportunities for, and barriers to, future convergence, and on ways to construct meaningful comparison across countries.

## 2 Methods

Our qualitative study collected and classified the indicators used by regulatory agencies in a sample of European countries to monitor the quality of hospital care in their jurisdictions.

### 2.1 Sample

We selected England, Germany, France, and the Netherlands; four neighbouring EU member states with advanced economies and similarly well-developed but differently structured systems of healthcare and varied governance traditions [37]. Their contrasting organisation of payers and providers within their respective healthcare systems and their distinctive regulatory arrangements might be expected to offer different opportunities for indicator construction and create different demands for quality measures. At the same time, all four countries have participated in the OECD Healthcare indicators project [6,28] and initiatives by the World Health Organization [38] and EU [39] to develop standardised quality measures of international health system effectiveness. There are, therefore, good institutional reasons to expect convergence beyond the universal desire to follow best practice in quality measurement.

International comparisons of how regulators in different countries monitor healthcare quality pose considerable methodological difficulties, not least because regulators often operate within complex and nationally distinctive landscapes of state and non-state organisations, such as medical professional associations, clinical disease registries, and insurers that have developed their own healthcare quality indicators for overlapping or different purposes [40]. In this paper, however, we address that problem of comparison by restricting our analysis to the official indicator sets used by the supervisory organisations charged by law with monitoring the quality of acute hospital healthcare in each country:

• **England:** Hospital care is almost entirely provided by the single-payer state-run NHS. Healthcare quality is overseen by the Care Quality Commission (CQC), which is a non-departmental public body responsible for regulating the quality of care by all health and social care providers. As well as licencing and inspecting providers, the CQC can issue regulatory improvement notices and put the management of poor quality hospitals into ‘special measures’ [41]. Its enforcement activities are based on inspection findings and analysis of the wealth of performance data routinely collected by NHS England and the Department of Health to inform the administration and financing of the NHS.
• **Germany:** Hospital care is delivered by public and private providers funded by para-public social insurance funds. Healthcare quality is overseen by the Gemeinsamer Bundesausschuss (G-BA), which is a Federal joint committee of medical professionals, social insurers, and healthcare providers operating independently of the Ministry of Health. The G-BA sets and monitors quality standards and determines which procedures and providers are eligible for reimbursement. The G-BA also works collaboratively to design quality indicators that draw on hospital quality assurance data collected and published by external contractors, which the G-BA uses to engage in ‘structured dialogue’ with providers if their performance deviates from pre-determined norms.
• **France:** Hospital care is likewise delivered by public and private providers largely funded by social insurance funds. Healthcare quality is overseen by the Haute Autorité de Santé (HAS), which is an independent administrative authority responsible for accrediting and certifying the quality of care provided by hospitals, clinics, and other health care facilities. HAS designs indicators in consultation with voluntary and independent health professionals and patients, conducts peer review visits of hospitals on a routine basis, collects data, and publishes assessment results online. Enforcement is left to regional health agencies, which were given responsibility for health and social care planning, regulation, and enforcement of national health policy priorities by the devolution laws of 2009.
• **The Netherlands:** Hospital care is delivered mostly by private not-for-profit foundations, which have been funded through mandatory, and strictly regulated, private insurance since 2006 [42]. Healthcare quality is overseen by the Inspectie Gezondheidszorg en Jeugd (IGJ), which is a government inspectorate within the Ministry of Health, Welfare and Sport, responsible for regulating the quality of health and social care, as well as youth services, and ensuring a level-playing field among providers. The IGJ executes those tasks by licencing, inspecting, and policing hospitals, using a pyramid of compliance tools and sanctions. The IGJ also publishes measures of the quality of care delivered by every Dutch hospital, based on hospital-reported data and indicators designed in collaboration with medical professional organizations and the hospital themselves.

### 2.2 Indicator definition and data sources

We define an indicator as a discrete variable providing some nominal, ordinal, or quantitative measure of healthcare quality. Indicators can either be a single measure, such as the number of ‘never events’ recorded in English hospitals (STEISNE in [43]), or they can combine multiple measures into a ‘composite indicator’, such as the French indicator for the quality of discharge records for psychiatric patients (TDP2 PSY in [44]) which aggregates together 15 discrete fields of information (TDP2 PSY1-15 in [44]).

In 2016 we compiled a database of the hospital quality indicators used at that time by our four supervisory agencies [[43], [44], [45], [46]]. Where indicator lists were published in English, as they were for Germany [45] and England [43], we used those; otherwise we worked with the original listings in Dutch [46] and French [44] and drew on a corpus of 32 background interviews conducted with key informants from the four countries to clarify any uncertainties about particular indicators and help explain differences in indicator selection and use between countries. To ensure consistency and capture the variety and granularity of quality measures, we decomposed ‘composite’ indicators into their constitutive ‘sub-indicators’. In the process we excluded purely administrative measures used to facilitate data collection, e.g. the Dutch measure “Does your hospital perform colorectal surgery?” (17.2.2 in [46]), or enable cross-tabulation of patient survey results by patient condition, e.g. the French measure “did you need help with routine activities (washing, dressing, eating, …)?” (E-SATIS20 in [44]).

In total, our dataset of disaggregated indicators comprises 1,100 indicators: England 226; Germany 431; France 260; Netherlands 183.

### 2.3 Conceptual framework for indicator classification

We used an iterative process of expert judgment to classify each indicator in our database in three different ways, which we summarise in Table 1 and describe at greater length in a methodological Appendix A (see the online-only Supplementary Material associated with this paper). First, we categorised each indicator according to the Donabedian distinction between structure, process, and outcome-based approaches to measuring quality [47].

**Table 1:** Conceptual categories for classifying each indicator in terms of its Donabedian style of measurement; the dimensions of quality it assesses; and hospital activities it oversees.

**Donabedian style of quality measurement**	**Dimensions of quality**	**Hospital department or activity**
**Outcome**: the effect of care on the health status and/or satisfaction of the patient with their treatment **Process**: how healthcare is delivered **Structure**: type and amount of financial, human, material or organisational resources used by a health care organization to deliver services	**Safety**: preventing adverse outcomes for patients arising from care intended to help them **Effectiveness**: efficacy of care in benefitting those who need it while avoiding unnecessary treatment **Patient-centredness**: responsiveness of care to patient values, preferences, and needs **Timeliness**: delays and other barriers in accessing appropriate care **Efficiency**: cost-effectiveness and productivity of providers in delivering care **Equity**: fairness and impartiality in healthcare distribution, delivery, and outcomes **Well documented**: accuracy, completeness, & security of administrative record-keeping about patients and their care **Trained & certified**: staff licencing, training and CPD up-to-date and appropriate	**A&E** **Anaesthesia** **Cardiology** **Gastroenterology** **Geriatrics** **Intensive care (ICU)** **Nephrology** **Neurology** **Obstetrics** **Oncology** **Orthopaedics** **Outpatient care** **Paediatrics** **Psychiatry** **Rehabilitation** **Respiratory medicine** **Other medical depts:** clinical specialties unique to an indicator set with ≤5 indicators (e.g. dermatology) **Hospital-wide:** indicators for clinical activities that span the hospital e.g.nursing,infection control **Non-clinical services:** e.g. catering, parking **Management:** e.g. finance, administration, and other oversight functions

Second, we assessed the dimension of quality being measured by each indicator, using the ‘dimensions of quality’ framework first developed by the Institute of Medicine (IoM) [5] and later elaborated by the World Health Organization and OECD [28,29], which added equity to the original IoM dimensions of safety; medical effectiveness; patient-centeredness; timeliness and access; and efficiency. To these conventional dimensions of quality, our analysis led us to conceptualise two further dimensions: ‘well documented’, for indicators assessing the quality of administrative paperwork, medical records, and information handover between clinicians; and ‘trained & certified’, for indicators measuring the training and skills of the hospital workforce.

Finally, we recorded the particular specialty or part of the hospital to which each indicator pertained, drawing on the list of medical specialties across EU member states set out in EU Directive 2005/36/EC (Annex V) on the recognition of professional qualifications and including non-clinical support services and management (see the online-only Supplementary Materials for further details).

### 2.4 Coding process

To ensure validity and reliability, each indicator was coded in four iterative steps. First, the four authors worked through a sample of indicators from each country to develop a consistent understanding of our classification categories. Second, three of the authors worked together to code each indicator in turn according to those categories. Third, the corresponding author then repeated the coding exercise independently. Finally, for the small number of indicators where conflicts in coding arose, reconciliation was achieved through in-depth discussion by all four authors until consensus was reached.

### 2.5 Limitations

Our study has at least three limitations. First, our datasets represent a snapshot in time. Indicator sets have continued to evolve, but as we explain in the ‘Discussion’ and ‘Conclusions’, there are good reasons to believe that further evolutionary developments are unlikely to affect the broad patterns of difference we observe between countries. Second, the process of coding involved a significant degree of subjectivity. However, that subjectivity is mitigated by our large N and our reconciliation processes, which increase the likelihood that even if our classification of any single indicator is uncertain and contestable, any individual coding errors are likely to cancel out within such a large dataset. Third, we have restricted our analysis to the official indicator sets used by the supervisory organisations charged by law with monitoring the quality of acute hospital healthcare in each country. Further research would be needed to analyse the various indicators used by other state and non-state organisations in each of our four case study jurisdictions.

## 3 Results

Supervisory agencies in our four countries each collected data for hundreds of hospital quality indicators, but not one of 1,100 indicators in their official sets was concerned with equity, despite the emphasis given to it in international comparisons of healthcare system performance [5,10,28,29]. Aside from this universal lacuna, the four national indicator sets differed substantially in their balance of Donabedian measurement styles, the dimensions of quality they considered, and the particular hospital activities they scrutinised. Those differences are shown in Table 2 below. We describe the distinctive patterns of indicator use in each country in the following sub-sections.

**Table 2:** Numbers of quality indicators used in 2016 by the English CQC, the German B-GA, the French HAS and the Dutch IGJ disaggregated by Donabedian style, quality dimension, and hospital activities.

	England	Germany	France	Netherlands
**Donabedian style**				
Structure	153	4	39	63
Process	12	119	145	90
Outcome	61	308	76	30

**Dimensions of quality**				
Safety	97	214	55	27
Effectiveness	51	208	23	60
Patient centredness	27	–	49	–
Timeliness	14	3	4	8
Efficiency	22	–	–	4
Equity	–	–	–	–
Well documented	5	6	123	77
Well certified	10	–	6	7

**Hospital department or activity**				
A&E	12	–	–	–
Anaesthesia	1	–	17	6
Cardiology	24	148	17	31
Gastroenterology	16	37	–	18
Geriatrics	–	–	–	17
Intensive care (ICU)	–	–	–	6
Nephrology	5	47	6	–
Neurology	9	18	9	12
Obstetrics	4	26	4	4
Oncology	3	8	1	30
Orthopaedics	23	102	–	9
Outpatient care	–	–	16	–
Paediatrics	5	25	–	19
Psychiatry	4	–	17	–
Rehabilitation	–	–	22	–
Respiratory medicine	8	17	–	3
Other medical depts	26	–	–	2
Hospital-wide	33	3	89	14
Non-clinical services	4	–	9	–
Management	49	–	53	12
**Total number of indicators**	**226**	**431**	**260**	**18**

### 3.1 England

**Fig. 1:**
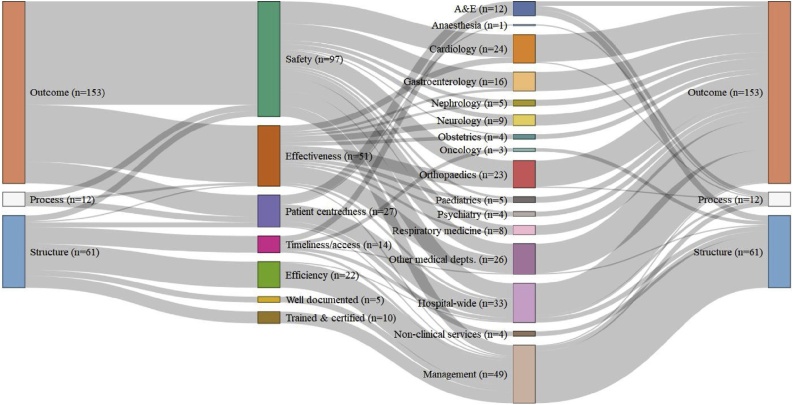
The Donabedian style, quality dimension, and hospital activities monitored by each quality indicator used in 2016 by the English CQC [43].

In England (Fig. 1), the CQC relied on outcome measures (68%), supplemented by some structure (27%) and process (5%) ones, to monitor a broad range of quality dimensions and hospital activities. Indicators were constructed from a huge variety of administrative data sources, including patient records, organisational reporting, complaints and whistle-blowing logs. Indicators also made use of surveys; both of patients, to get at patient-centredness, and of staff, whose responses were used for a variety of structure and process indicators, such as the proportion of staff experiencing physical violence (COM_ABUSESTA in [43]) or judging incident reporting procedures to be fair and effective (NHSSTAFF11 in [43]). Hospital standardised mortality rates (HSMRs), which are risk-adjusted to take account of varying patient mix, provided the CQC with the vast majority (85%) of its safety indicators. However, HSMRs, only provide a plurality (43%) of the many different kinds of outcomes the CQC monitored, which included emergency readmission rates and patient-reported outcome measures of safety and medical effectiveness as well as various survey-derived measures of patient-centredness. This latter quality, along with waiting times and the efficiency with which hospitals manage the public resources provided to them in England’s single-payer healthcare system, were major concerns for the CQC. Indeed, the CQC had more indicators devoted to monitoring efficiency and hospitals’ ability to deliver timely care than our other three countries put together.

CQC indicators provided a synoptic overview of care quality across the hospital, covering 22 different hospital specialities, including 10 we combined together as ‘other medical departments’ because they were few in number and unique to England. As will become clear below, the scope of English hospital monitoring was far wider than in other countries, but also comparatively shallow. Most notably, the CQC’s outcomes-focus tended to preclude much scrutiny of the processes of delivering particular kinds of care. Just two of its 226 indicators measured compliance with best practice guidance (‘proportion of patients receiving all secondary prevention medication for which they are eligible’ (MINAP22 in [43]); ‘proportion of cases complying with all nine standards of care set out by the National Hip Fracture Database’ (NHFD01 in [43]). Rather than auditing clinical governance processes, the CQC focused instead on hospital management and on various hospital-wide indicators of quality, like waiting times, nosocomial infection, and re-admission rates, as well as patient satisfaction with non-clinical services, like catering and housekeeping.

### 3.2 Germany

**Fig. 2:**
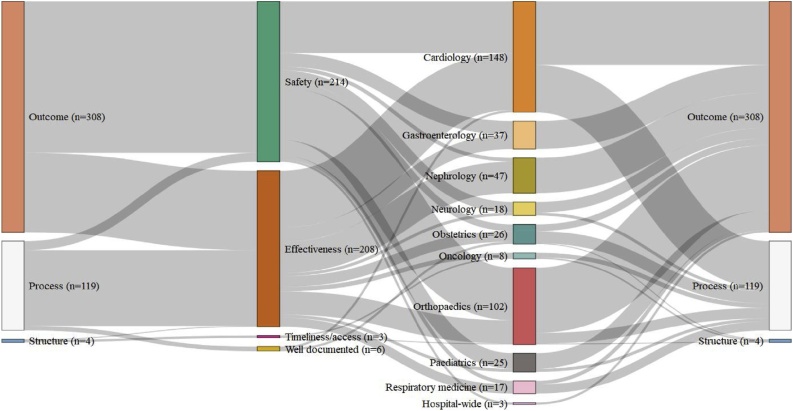
The Donabedian style, quality dimension, and hospital activities monitored by each quality indicator used in 2016 by the German G-BA [45].

In Germany, hospital quality indicators focused almost entirely on the safety and medical effectiveness of a few, largely surgical, interventions (Fig. 2). The G-BA’s indicator set was composed primarily of outcome measures (71 %). These were derived from mandatory hospital reporting of discrete outcomes from particular interventions, such as raw rates of mortality and inability to walk at discharge after knee replacement surgery (QI-ID 2277 and 2272 in [45]), rather than from administrative payment data or individual patient records, which might have been used to risk-adjust measures of hospital performance by taking account of varying patient mix. Process indicators focused largely on medical effectiveness by compliance with best practice guidelines, such as the number of hip replacement surgeries fulfilling indication criteria (QI-ID 1082 in [45]). The G-BA’s handful of structure measures related to the availability of paediatricians at premature births and delays between diagnosis and surgery, reflecting the German concern with ensuring clinical excellence [48], rather than efficiency or patient centredness, which were not otherwise monitored.

This clinical orientation was also reflected in the focus of the German indicator set on intensively monitoring a limited range of largely surgical interventions, rather than considering quality at the broader hospital-level. Indeed, the hospital as an organisational entity hardly figured in the G-BA’s quality monitoring framework. Over a third (34 %) of its indicators focused on a single specialty—cardiology-- for which there were 148 indicators measuring the safety and effectiveness of particular surgical procedures, such as pacemaker implantation and heart transplants. Likewise, the focus of orthopaedic, nephrology, and gastroenterology indicators was also on surgical interventions rather than other kinds of treatment delivered by those specialities. The only non-surgical quality indicators were for obstetrics and the 17 indicators for the treatment of community-acquired pneumonia (classified as ‘respiratory medicine’ in Fig. 2). Beyond these specialties, there were only 3 indicators for hospital-wide aspects of clinical care, such as nursing, and no indicators for non-clinical services or hospital management.

### 3.3 France

**Fig. 3:**
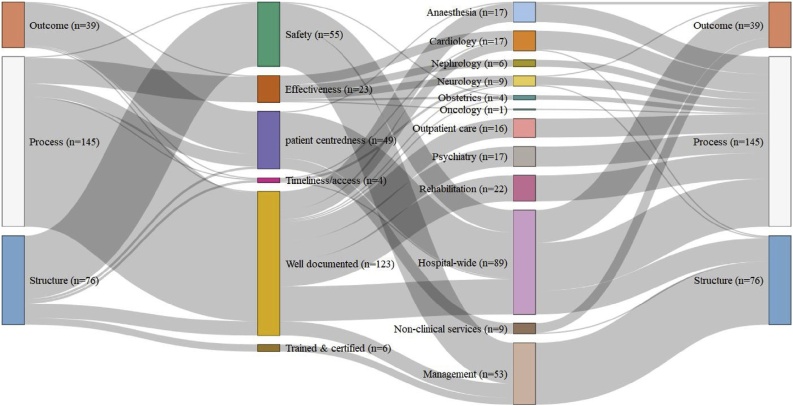
The Donabedian style, quality dimension, and hospital activities monitored by each quality indicator used in 2016 by the French HAS [44].

In contrast to England and Germany, the French indicator set consisted of mostly structure and process measures (Fig. 3), which HAS constructed from hospital reporting and auditing randomly sampled patient files. There were just two clinical outcome indicators-- for post-operative pain-level and autonomy after discharge from stroke (DAN EVA and AVC9 in [44])-- and none of the mortality indicators so common in England and Germany, with the first HSMR (for myocardial infarction) still under development and not set for release until 2020 [49]. Instead, outcome measures in France were almost entirely concerned with patient experiences of care, captured through survey questions about, *inter alia,* pain relief (E-SATIS29 in [44]), parking (E-SATIS1 in [44]), and the welcome provided by administrative staff (E-SATIS2 in [44])). In the absence of objectively measurable indicators of clinical outcomes, safety was assessed through structural measures of whether hospitals had appropriate protocols for managing nosocomial infections, while effectiveness was largely captured through process measures of adherence to protocols for assuring the quality of care, such as prescription of beta-blockers to heart attack patients on release from hospital (BBL in [44]). In this way, quality in France was treated as a function of hospital organisation rather than the skill of individual clinicians. Indeed, almost half of French indicators were process measures of the quality of medical record-keeping, *de facto* linking good medical practice to the paperwork needed to support the patient journey through the healthcare system.

This organisational approach to healthcare quality in France was also reflected in the emphasis given to monitoring general hospital functions. The majority (67 %) of French indicators focused on aspects of performance across the hospital, including non-clinical services like catering as well as various clinical functions, such as pain relief, patient rehab, and -most notably- nosocomial infection control, which was the subject of more than a quarter of all indicators. However, with just a few exceptions, such as psychiatry, for which France had many more indicators than any other country, much less attention was paid to monitoring individual medical specialities or interventions.

### 3.4 Netherlands

**Fig. 4:**
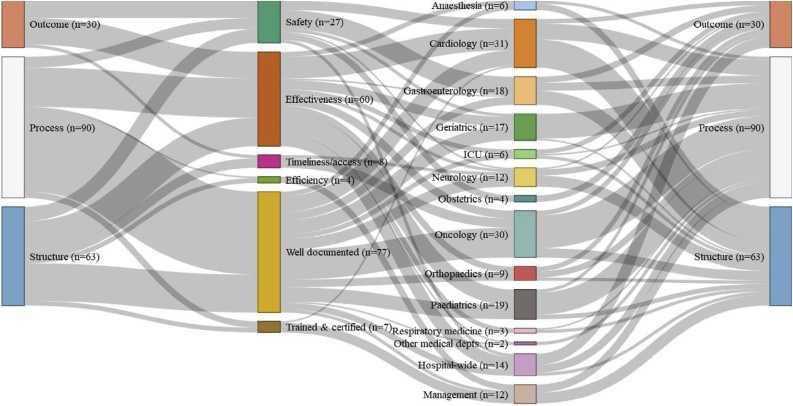
The Donabedian style, quality dimension, and hospital activities monitored by each quality indicator used in 2016 by the Dutch IGJ [46].

In the Netherlands, the IGJ drew exclusively on mandatory hospital reporting to construct its own collaboratively designed indicator set (Fig. 4). The set predominantly comprised process (49 %) and structure (34 %) indicators, many of which, like in France, focused on the quality of documentation. For the IGJ, however, ‘well documented’ measured hospital participation in various specialty-based national registries, like the percentage of eligible operations registered with the Dutch Spine Surgery Registry (1.5.1 in [46]), rather than the quality of individual patient records, as in France. The Dutch principally assessed safety and medical effectiveness through a clinical governance focus on structure and process measures of adherence to best clinical practice, supplemented by various patient-reported outcome measures. However, unlike the CQC and G-BA, the IGJ indicator set included raw mortality indicators for just two interventions, only one of which was then risk-adjusted. Nor did the IGJ make use of patient-survey based indicators, which were instead collected by the Dutch National Health Care Institute (ZIN) to help patients choose their provider [50].

The IGJ monitored a wide range of services across the hospital. With indicators for 13 discrete functions, including two (minimally invasive surgeries [1.7.1 in [46]], and diabetic foot ulcers [1.8.1 in [46]]) that we classified under ‘other medical departments’, it was second only to the CQC in England in the number of clinical specialities it monitored. There were a few indicators about hospital-wide issues like nursing care and human resource management, but they also had a clinical focus. There were no indicators for non-clinical services and the vast majority (86 %) of indicators focused on the quality of particular specialties that patients might choose, like in Germany, rather than on the hospital as an organisational unit, like in France.

## 4 Discussion

Despite the universal desire to monitor healthcare quality and substantial international efforts to identify and share best practice in measuring it, our cross-country comparison reveals striking differences in the official indicator sets used by statutory regulators to monitor the quality of hospital care in England, Germany, France, and the Netherlands.

One way in which official indicator sets differed was in their use of structure, process, and outcome indicators. Germany stood out for almost entirely eschewing structure measures in favour of outcome and process ones. By contrast, regulators in the other countries used all three indicator types more freely, with outcome indicators predominating in England, process indicators in France, and Dutch indicators evenly divided between Donabedian’s three styles of measurement.

Official indicator sets also differed in which dimensions of quality were monitored and how they were measured. Medical effectiveness and safety received universal attention, but apart from Germany — which focused almost exclusively on those two dimensions of quality — other countries had various additional quality concerns as well. Hospital record-keeping accounted for a third of Dutch indicators and half of French ones but was largely ignored in England. Patient experience was closely monitored by regulators in France and England but not by their Dutch or German counterparts. Likewise, efficiency was a concern in England and to a lesser extent in the Netherlands, but not in France or Germany.

Even when they monitored the same quality dimensions, regulators often defined and measured them in quite different ways. For example, more than 90 % of all safety indicators in both England and Germany were patient-reported outcomes, which they calculated in very different ways. While the CQC overwhelmingly relied on HSMRs, the G-BA measured a broader range of adverse clinical outcomes but did not standardise them to take account of hospitals’ varying patient mix and measure relative performance. In contrast to that outcome-focus, HAS assessed hospital safety in France by checking for the existence of clinical protocols to prevent hospital-acquired infections. The Dutch IGJ measured safety in the most diverse ways, including: checking the existence of, and compliance with, hospital infection controls and other speciality-specific safety protocols; measuring patient volumes to ensure surgeons were sufficiently practiced to be safe; calculating hospital standardised emergency readmission and complication rates, but almost no mortality rates.

Official indicator sets also focused on different kinds of hospital activity. German indicators intensively monitored a small number of largely surgical interventions, almost completely ignoring other kinds of medical care or the hospital itself as an organisation. By contrast, indicators in the other three countries covered a broader range of clinical specialities and were more concerned with hospital-wide processes and management. England monitored by far the widest set of hospital activities, while France was most pre-occupied by management of hospital-wide concerns, such as infection control and catering. The Dutch indicator set was concerned with how well hospital specialists cooperated with various national disease-based registries to support those registries’ quality improvement activities.

These findings are consistent with comparative health policy studies that have highlighted how new policy instruments are shaped by country-specific demands and constraints of national healthcare systems and governance traditions, as well as the interests and veto power of key actors, public preferences, and the structure of the wider political system [[51], [52], [53]]. Such factors are likely to create path dependencies in the way that quality indicators are developed and put to use in each country [8,[54], [55], [56]]. We can go further in explaining the nationally specific character of indicator sets, however, if we differentiate between ‘demand-side’ pressures for quality indicators, and ‘supply-side’ constraints on how indicators can be constructed.

‘Demand-side’ pressures help explain how national indicator sets ended up targeting such divergent dimensions of quality and hospital activities as they have responded to the distinctive policy problems emerging in each country’s healthcare system. Thus, the indicator set for England’s NHS was synoptic in its coverage of quality dimensions and hospital activities, because the state is responsible for everything: funding and delivering healthcare as well as regulating its quality. In this context, competing public demands for safe, speedy, and yet also inexpensive care have fuelled regular political crises. In response, politicians have charged the regulator — the CQC — with an ever-expanding list of quality concerns that its indicators must somehow monitor [9].

By contrast, official indicator sets in Germany, France, and the Netherlands were less comprehensive in their coverage of quality dimensions and hospital activities because in those social and private insurance systems the state is less immediately accountable for healthcare and so has left some matters to the healthcare sector. In Germany, the G-BA is relatively insulated from political pressures and has, therefore, been slow to expand the narrow scope of its indicator set, which was first introduced to prevent providers from compromising the safety and effectiveness of fixed price surgical procedures [57]. In France, HAS initially adopted a light touch to monitoring quality, restricting itself to patient experience surveys and assessing the quality of paperwork to guard against discontinuities in care by doctors operating in private practice within French traditions of liberal medicine [58,59]. However, oversight expanded in 2006 when a public crisis over nosocomial infections [60,61] prompted the state to develop safety indicators for infection control, giving France more than three times as many such indicators as the other countries put together. In the Netherlands, IGJ indicators have focused more on the clinical effectiveness and safety of discrete specialities, not least to ensure that the market-oriented healthcare reforms of 2006, which sought efficiency gains through managed competition, did not result in a race to the bottom on quality [62].

‘Supply-side’ explanations for indicator variety concern the way in which the configuration of national healthcare, political, and regulatory systems constrains the kinds of data that regulators can use for indicator construction [8]. As such they principally shape the balance between Donabedian measurement styles in each countries’ indicator set. Thus, in England’s pluralist political system, where the state is not bound to secure the agreement of competing stakeholder interests, the quasi-independent CQC has been free to construct indicators from the vast quantities of administrative data on NHS structures, performance, and patient outcomes that the state already collects routinely in discharging its responsibility for both the financing and provision of NHS healthcare. Accordingly the CQC indicator set was wide-ranging and in keeping with British commitments to ‘risk-based’ regulation [9,63], it used z-scoring techniques to highlight hospitals posing the greatest risk to quality standards, which were defined relatively in terms of statistical deviation from the mean rather than according to any absolute minimum standards [64].

However, comparable administrative data is less readily available in the social and private insurance systems of Germany, France and the Netherlands, not least because payment systems vary between insurers (despite ongoing efforts to standardise according to diagnostic-related groups [57]). Consequently, regulators have had to negotiate consistent reporting standards with sometimes reluctant, and often private, providers or otherwise source their own data in the face of varying legal and institutional constraints that are deeply rooted within their particular political systems and constitutional settings.

Thus, in Germany’s fragmented healthcare system indicator construction is highly constrained by both technical data availability and political restrictions on its use. Inconsistencies in patient recording systems make it difficult to take account of hospitals’ varying patient mix [57], which is one reason why mortality and other clinical outcome measures are not risk-adjusted, and hospital performance is benchmarked against absolute reference values rather than relatively as in England. Indicator development is also constrained by the federal political system, which makes the 16 state (Länder) governments — and not the B-GA — responsible for organisational aspects of hospital provision that might otherwise be served by structural indicators. Further constraints are created by the political need for corporatist consensus, and strong constitutional protections of business rights to economic activity [65], which open indicator design to legal challenge and were central to German hospitals successfully contesting minimum volumes regulation [66].

In France’s centralised pluralist political system, the government faces fewer political and constitutional constraints on data usage, but its fragmented healthcare system has forced HAS to collect much of its own indicator data, largely through auditing patient medical records, mandatory hospital reporting, and patient surveys. Those data sources have favoured structure and process measures and a concern with the quality of documentation and patient experience rather than clinical outcome measures, such as mortality indicators, which France was slow in developing, because of medical professional scepticism about unadjusted mortality rates [60,61]. HAS is currently developing its first HSMR, which is now possible thanks to a new database, the Système National des Données de Santé (SNDS) [67], which the state is creating to help with cost control by linking previously separate payment data with clinical in- and out-patient activity records and a national cause of death registry.

In the Netherlands, indicator selection and construction are less constrained by the technical challenges of data sourcing and linkage that trouble Germany and France, not least because the 2006 health care reforms required standardisation of payment data to ensure equitable distribution of the pool of high-risk patients. Rather the main constraint has been political insofar as the Dutch corporatist governance tradition means any new indicators must gain the consent of the various medical professional and hospital associations [8]. Process and structure indicators – which account for half and a third of the indicator set respectively— are widely accepted amongst stakeholders. By contrast, outcome indicators, which are widely used by clinical registries to support quality improvement initiatives, account for only a sixth of the official indicators used by the IGJ. It explains this imbalance by noting that “the reporting burden on an outcome indicator is much greater than a structure indicator” [46], but informally it is also clear that the IGJ uses outcome measures sparingly because their wider utility for regulatory purposes is not universally accepted [68]. Similarly, the IGJ indicator set does not include any patient survey-based measures because the patient experience is regarded as more relevant to informing patient choice — and thus the responsibility of ZIN — than quality assurance and regulation.

## 5 Conclusions

Our research on the use of quantitative indicators by healthcare quality regulators in four neighbouring European states shows that they define, measure, and monitor the quality of acute hospital care in starkly different ways. However, we go beyond the banal observation that countries have their own ways of doing things, much like they have different national flags. Rather we argue that contrasting indicator set designs reflect fundamental differences in national regulatory priorities, institutional configurations of payers and providers, and even understandings of quality itself. Although national indicator sets will continue to evolve, the patterns we identify here are likely to persist. That path-dependence reflects distinctive ‘demand-side’ pressures shaping the particular dimensions of quality and hospital activities targeted by national indicator sets, as well as ‘supply-side’ constraints on data availability and access shaping the Donabedian measurement styles adopted in different healthcare and regulatory systems.

Our analysis helps explain why international efforts to benchmark hospital quality and identify universal measures are so difficult [26,54]. In the absence of universal agreement about the meaning of quality, countries necessarily steer by their own lights when selecting quality indicators. Nevertheless, our analysis does suggest that international benchmarking could be made more tractable by looking for families of countries with similarly structured healthcare systems and governance traditions, where supply-side constraints on, and demand-side pressures for, measuring healthcare quality are better aligned.

## Contributors

All four authors co-conceived the study, conducted background interviews, analysed the data, and contributed to writing the article.

## Funding

The Economic and Social Research Council (ES/K006169/1) and the 10.13039/100010269Wellcome Trust (210346/Z/18/Z).

## Ethics

Ethical approval was obtained for the two projects under which this study was conducted from the Research Ethics Committee of King’s College London (REP(GSSHM)/13/14-5, MRA-17/18-5908).

## Declaration of Competing Interest

AG worked at the CQC up until 2016 and now works for Statica Research with a focus on patient feedback. DD, HR and A-LB report a grant from the ESRC (detailed above) and DD and HR report a grant from the Wellcome Trust (detailed above) during the conduct of the study

## Footnotes

Appendix A Supplementary material related to this article can be found, in the online version, at doi:https://doi.org/10.1016/j.healthpol.2020.02.012.

## References

[1] Thomson W. (1889). Popular lectures and addresses.

[2] Donabedian A. (1966). Evaluating the quality of medical care. The Milbank Memorial Fund Quarterly.

[3] Bodenheimer T. (1999). The movement for improved quality in health care. New England Journal of Medicine.

[4] Berwick D.M. (2016). Era 3 for medicine and health care. JAMA.

[5] Institute of Medicine (2001). Envisioning the national health care quality report.

[6] Mattke S., Epstein A.M., Leatherman S. (2006). The OECD health care quality indicators project: history and background. International Journal for Quality in Health Care.

[7] Fekri O., Macarayan E.R., Klazinga N. (2018). Health system performance assessment in the WHO european region: which domains and indicators have been used by member states for its measurement?. http://www.ncbi.nlm.nih.gov/books/NBK519096/.

[8] Pollitt C., Harrison S., Dowswell G., Jerak-Zuiderent S., Bal R. (2010). Performance regimes in health care: institutions, critical junctures and the logic of escalation in England and the Netherlands. Evaluation.

[9] Beaussier A.-L., Demeritt D., Griffiths A., Rothstein H. (2016). Accounting for failure: risk-based regulation and the problems of ensuring healthcare quality in the NHS. Health, Risk & Society.

[10] Blumenthal D., Malphrus E., McGinnis J.M. (2015). Vital signs: core metrics for health and health care progress. Vital signs: core metrics for health and health care progress.

[11] Burstin H., Leatherman S., Goldmann D. (2016). The evolution of healthcare quality measurement in the United States. Journal of Internal Medicine.

[12] DoH (2008). High quality care for all: NHS next stage review final report.

[13] Meyer G.S., Nelson E.C., Pryor D.B., James B., Swensen S.J., Kaplan G.S. (2012). More quality measures versus measuring what matters: a call for balance and parsimony. BMJ Quality Safety.

[14] Health Quality Ontario (2019). A sustainable Indicator reduction and management strategy for the Ontario hospital sector. https://www.hqontario.ca/Portals/0/documents/system-performance/oha-sustainable-indicator-reduction-and-management-strategy-en.pdf.

[15] Rubin H.R., Pronovost P., Diette G.B. (2001). From a process of care to a measure: the development and testing of a quality indicator. International Journal for Quality in Health Care.

[16] Spiegelhalter D.J. (2005). Funnel plots for comparing institutional performance. Statistics in Medicine.

[17] De Vos M., Graafmans W., Kooistra M., Meijboom B., Van Der Voort P., Westert G. (2009). Using quality indicators to improve hospital care: a review of the literature. International Journal for Quality in Health Care.

[18] Powell A.E., Davies H.T.O., Thomson R.G. (2003). Using routine comparative data to assess the quality of health care: understanding and avoiding common pitfalls. BMJ Quality & Safety.

[19] Jarman B., Gault S., Alves B., Hider A., Dolan S., Cook A. (1999). Explaining differences in English hospital death rates using routinely collected data. BMJ.

[20] Rothberg M.B., Morsi E., Benjamin E.M., Pekow P.S., Lindenauer P.K. (2008). Choosing the best hospital: the limitations of public quality reporting. Health Affairs.

[21] Austin J.M., Jha A.K., Romano P.S., Singer S.J., Vogus T.J., Wachter R.M. (2015). National hospital ratings systems share few common scores and may generate confusion instead of clarity. Health Affairs.

[22] Griffiths A., Leaver M.P. (2018). Wisdom of patients: predicting the quality of care using aggregated patient feedback. BMJ Quality Safety.

[23] Griffiths A., Beaussier A.-L., Demeritt D., Rothstein H. (2017). Intelligent Monitoring? Assessing the ability of the Care Quality Commission’s statistical surveillance tool to predict quality and prioritise NHS hospital inspections. BMJ Quality Safety.

[24] Veillard J., Moses McKeag A., Tipper B., Krylova O., Reason B. (2013). Methods to stimulate national and sub-national benchmarking through international health system performance comparisons: a Canadian approach. Health Policy.

[25] Basu S., Andrews J., Kishore S., Panjabi R., Stuckler D. (2012). Comparative performance of private and public healthcare systems in low- and middle-income countries: a systematic review. PLOS Medicine.

[26] Papanicolas I., Jha A.K. (2017). Challenges in international comparison of health care systems. JAMA.

[27] Shahian D.M., Normand S.-L.T., Friedberg M.W., Hutter M.M., Pronovost P.J. (2016). Rating the raters: the inconsistent quality of health care performance measurement. Annals of Surgery.

[28] Arah O., Westert G., Hurst J., Klazinga N. (2006). Conceptual framework for the OECD health care quality indicators project. International Journal for Quality in Health Care.

[29] World Health Organization (‎2007). Regional Office for Europe. Performance Assessment Tool for Quality Improvement in Hospitals (‎PATH).

[30] European Commission (2020). Expert Group on Health System Performance Assessment (HSPA). https://ec.europa.eu/health/systems_performance_assessment/policy/expert_group_en.

[31] Järvelin J., Häkkinen U. (2012). Can patient injury claims be utilised as a quality indicator?. Health Policy.

[32] Perera R., Dowell A., Crampton P. (2012). Painting by numbers: a guide for systematically developing indicators of performance at any level of health care. Health Policy.

[33] Berg M., Meijerink Y., Gras M., Goossensen A., Schellekens W., Haeck J. (2005). Feasibility first: developing public performance indicators on patient safety and clinical effectiveness for Dutch hospitals. Health Policy.

[34] Groene O., Skau J.K.H., Frølich A. (2008). An international review of projects on hospital performance assessment. International Journal for Quality in Health Care.

[35] Bramesfeld A., Wensing M., Bartels P., Bobzin H., Grenier C., Heugren M. (2016). Mandatory national quality improvement systems using indicators: an initial assessment in Europe and Israel. Health Policy.

[36] Schweppenstedde D., Hinrichs S., Ogbu U., Schneider E.C., Kringos D.S., Klazinga N.S. (2014). Regulating quality and safety of health and social care. Rand Health Quarterly.

[37] (2017). Country profiles : international health care system profiles. http://international.commonwealthfund.org/countries/.

[38] Carinci F., Van Gool K., Mainz J., Veillard J., Pichora E.C., Januel J.M. (2015). Towards actionable international comparisons of health system performance: expert revision of the OECD framework and quality indicators. International Journal for Quality in Health Care.

[39] Expert Group on Health Systems Performance Assessment (2016). So what? Strategies across Europe to assess quality of care. https://ec.europa.eu/health/sites/health/files/systems_performance_assessment/docs/sowhat_en.pdf.

[40] Klazinga N. (2000). Re-engineering trust: the adoption and adaption of four models for external quality assurance of health care services in western European health care systems. International Journal for Quality in Health Care.

[41] Monitor. A Guide to Special Measures (Updated February 2015). Available from: https://www.gov.uk/government/uploads/system/uploads/attachment_data/file/403083/Special_measures.

[42] van de Ven W.P.M.M., Schut F.T. (2008). Universal Mandatory Health Insurance In The Netherlands: A Model For The United States?. Health Affairs.

[43] CQC (2015). Intelligent Monitoring: NHS acute hospitals indicators and methodology—may 2015.

[44] HAS (2020). Indicateurs de qualité et de sécurité. https://www.has-sante.fr/portail/jcms/fc_1249986/fr/indicateurs-de-qualite-et-de-securite.

[45] AQUA (2015). German hospital quality report, 2013.

[46] IGZ. Basisset kwaliteitsindicatoren ziekenhuizen (2016). Inspectie voor de Gezondheidszorg, 2015. https://www.igj.nl/binaries/igj/documenten/indicatorensets/2015/08/06/basisset-kwaliteitsindicatoren-ziekenhuizen-2016/IGZ+Basisset+kwaliteitsindicatoren+ziekenhuizen+2016_tcm294-367407.pdf.

[47] Donabedian A. (1988). The quality of care: how can it be assessed?. JAMA.

[48] Altenhofen L., Birkner B., Blumenstock G., Geraedts M., Gibis B., Jäckel W. (2005). Qualitätsindikatoren in Deutschland. https://www.aezq.de/mdb/edocs/pdf/literatur/qi-positionspapier.pdf.

[49] HAS (2017). Indicateurs de mortalité hospitalière : expériences étrangères, enseignements de la littérature et recommandations pour l’aide à la décision publique et le développement d’indicateurs en France. https://www.has-sante.fr/upload/docs/application/pdf/2017-10/rapport_mortalite_2017.pdf.

[50] Delnoij D.M., Rademakers J.J., Groenewegen P.P. (2010). The Dutch Consumer Quality Index: an example of stakeholder involvement in indicator development. BMC Health Services Research.

[51] Wilsford D. (1994). Path dependency, or why history makes it difficult but not impossible to reform health care systems in a big way. Journal of Public Policy.

[52] Tuohy C.H. (1999). Accidental logics: the dynamics of change in the health care arena in the United States, Britain, and Canada.

[53] Moran M. (1999). Governing the health care state: a comparative study of the United Kingdom, the United States, and Germany. Manchester.

[54] Oliver A. (2012). The folly of cross-country ranking exercises. Health Economics, Policy and Law.

[55] Kodate N. (2010). Events, public discourses and responsive government: quality assurance in health care in England, Sweden and Japan. Journal of Public Policy.

[56] Örnerheim M., Triantafillou P. (2016). Explaining quality management in the Danish and Swedish public health sectors: unintended learning and deliberate co-optation. International Journal of Public Administration.

[57] Busse R., Nimptsch U., Mansky T. (2009). Measuring, monitoring, and managing quality in Germany’s hospitals. Health Affairs.

[58] Porras-Gallo M.-I. (2007). Between the German model and liberal medicine: the negotiating process of the state health care system in France and Spain (1919–1944). Hygiea Internationalis.

[59] Rodwin V.G. (2003). The health care system under French national health insurance: lessons for health reform in the United States. American Journal of Public Health.

[60] Bertillot H. (2016). Des indicateurs pour gouverner la qualité hospitalière. Sociogenèse d’une rationalisation en douceur. Sociologie du Travail.

[61] Weckert E., Bertillot H. (2014). La régulation de la qualité dans le secteur de la santé. Quaderni. Communication, Technologies, Pouvoir.

[62] Helderman J.-K., Schut F.T., van der Grinten T.E.D., van de Ven W.P.M.M. (2005). Market-oriented health care reforms and policy learning in the Netherlands. Journal of Health Politics, Policy and Law.

[63] Demeritt D., Rothstein H., Beaussier A.-L., Howard M. (2015). Mobilizing risk: explaining policy transfer in food and occupational safety regulation in the UK. Environment and Planning A.

[64] Spiegelhalter D., Sherlaw-Johnson C., Bardsley M., Blunt I., Wood C., Grigg O. (2012). Statistical methods for healthcare regulation: rating, screening and surveillance. Journal of the Royal Statistical Society: Series A (Statistics in Society).

[65] Rothstein H., Borraz O., Huber M. (2013). Risk and the limits of governance: exploring varied patterns of risk-based governance across Europe. Regulation & Governance.

[66] Ettelt S. (2017). The politics of evidence use in health policy making in Germany: the case of regulating hospital minimum volumes. Journal of Health Politics, Policy and Law.

[67] Scailteux L.-M., Droitcourt C., Balusson F., Nowak E., Kerbrat S., Dupuy A. (2019). French administrative health care database (SNDS): the value of its enrichment. Therapies.

[68] Moes F.B., Houwaart E.S., Delnoij D.M.J., Horstman K. (2019). Strangers in the ER”: quality indicators and third party interference in Dutch emergency care. Journal of Evaluation in Clinical Practice.

