# Supplementary material for: Steering by their own lights: Why regulators across Europe use different indicators to measure healthcare quality
Source: Health Policy. 2020 May;124(5):501–10. doi: 10.1016/j.healthpol.2020.02.012 (PMC7677115; doi:10.1016/j.healthpol.2020.02.012)
Supplement: Supplementary file 1 [file mmc1.docx]

# METHODOLOGICAL APPENDIX

We classified each indicator in our database in three different ways. First, we categorised each indicator according to the Donabedian distinction between structure, process, and outcome-based approaches to measuring quality (See Table A) [1]. S*tructure indicators* measure the type and amount of financial, human, material and organisational resources used by health care organizations to deliver services. By contrast, *process indicators* measure the delivery of appropriate (or inappropriate) healthcare to the relevant population, consistent with best current professional knowledge. Lastly, *outcome indicators* measure the effect of care on the health status of the patient as well as improvement in patient knowledge, behaviour and satisfaction with their treatment.

**Table A. Donabedian style of quality measurement**

| Structure | Type and amount of financial, human, material or organisational resources used by a health care organization to deliver services |
| --- | --- |
| Process | How healthcare is delivered |
| Outcome | The effect of care on the health status and/or satisfaction of the patient with their treatment |

Though clear in theory, Donabedian’s threefold model was not always easy to apply in practice. One ambiguity concerned the classification of indicators measuring the existence and application of various care protocols. Indicators assessing a hospital’s adoption of protocols, for example to deal with nosocomial infections, were classified as structure indicators, insofar as they were about the resources available to a hospital in delivering care. By contrast, indicators measuring how well protocols were followed in practice, were classified as process indicators. Another ambiguity concerned the classification of outcome indicators that were measuring outcomes for organisations rather than Donabedian’s classic definition of outcome measures as pertaining to “the effects of care on the health status of patients and populations” [1]. We classified indicators addressing organisational outcomes, such as the CQC’s use of the Monitor risk rating of the financial health and governance of an NHS trust (MONITOR01 in [2]), as structure indicators insofar as they were concerned more with the structure and inputs to care than outcomes for patients.

Second, we classified each indicator in terms of the regulatory goals it was assessing, using the ‘dimensions of quality’ framework [3]. This basic approach to conceptualising quality was pioneered by the Institute of Medicine (IoM) [3], which identified five dimensions of quality-- *safety*; *effectiveness*; *patient-centeredness*; *timeliness and access*; *efficiency*-- as “definable, preferably measurable, attributes of the system that are related to its functioning to maintain, restore, or improve health”[4]. This framework was further elaborated by the World Health Organization and OECD [5,6], which added equity as another distinct dimension of healthcare quality that indicators might attempt to capture.

Applying this basic framework to our indicators required some further elaboration to define those different dimensions of quality more closely. Differentiating between safety and effectiveness could be particularly challenging. For example, should indicators about mortality among very small pre-term infants be classified as medical effectiveness, since survival is a key measure of the effectiveness of neonatal care, or of safety, insofar as the first duty of the physician is to do no harm? Faced with such borderline cases, we categorised those indicators dealing especially with the harm done to patients during an episode of care (for instance, in-hospital mortality or nosocomial infections following a procedure) as safety. In turn, we treated indicators about longer-term outcomes of care (for instance survival rates after x years for heart transplant and per cent of patients readmitted after a procedure) as effectiveness. The notions of *harm, directness and immediacy* were our major criteria for differentiating between these two dimensions of quality. Using a similar logic, we classified ‘volume’ indicators dealing with the number of patients treated for a specific condition and with a specific procedure (for instance volume of bariatric surgery performed in a year) as indicators of safety or effectiveness rather than access, because professional guidelines often recommend a minimum volume of operations be performed each year in order to ensure the skill of the team and hence the safety of the procedure [7].

A number of indicators did not fit the conventional dimensions of quality described by the IoM, WHO, and OECD, which forced us to invent two additional quality dimensions. In particular, we observed that many indicators, especially in France and the Netherlands, were concerned with measuring the quality of the administrative paperwork, medical records, and information handover to relevant professional or administrative organisations. To capture this regulatory goal, we created an additional dimension of quality we termed “*well documented*”. Another focus that conventional dimensions of quality failed to capture was the concern, especially in England and the Netherlands, with assessing the training and skills of the hospital workforce. To capture this regulatory desideratum, we created an additional category we called “*trained and certified*”, giving us a total of eight dimensions of quality for classifying each indicator in our dataset (Table B).

**Table B. Dimensions of quality used in our** **classification**

| Safety | preventing adverse outcomes for patients arising from care intended to help them |
| --- | --- |
| Medical effectiveness | efficacy of care in benefitting those who need it while avoiding unnecessary treatment |
| Patient-centeredness | responsiveness of care to patient values, preferences, and needs |
| Timeliness | delays and other barriers in accessing appropriate care |
| Efficiency | cost-effectiveness and productivity of providers in delivering care |
| Equity | fairness and impartiality in healthcare distribution, delivery, and outcomes |
| Well documented | accuracy, completeness, & security of administrative record-keeping patient care and associated clinical processes |
| Well-trained and certified | staff licensing, training and continuing professional development up-to-date and appropriate |

Third, we sought to identify the particular specialty or hospital activity to which a given indicator pertained with a field we term “hospital department or activity”. This classification exercise was complex, as there are many different ways to classify hospital activities, so we adopted an iterative approach to refining this field. We began by considering the series of occupation codes describing divisions of clinical work in the British NHS, which may be defined by body systems (e.g. dermatology), patient demography (e.g. paediatrics), clinical technology (e.g. nuclear medicine), clinical function (e.g. rheumatology), disease type (e.g. oncology) or combinations of these factors. This list of recognised specialities in the NHS is tied to the EU directive 2005/36/EC on the recognition of professional qualifications, whose Annex V lists the equivalences for various medical specialties in different EU member states, on which we based our classification.

We further refined that very long list of specialities by comparing it against the major section headings in each indicator set to identify some commonly used designations like ‘oncology’, ‘cardiology’, and ‘psychiatry’. Specialities for which only one country maintained an indicator and the total number of such indicators was less than or equal to five were consolidated into a category we termed ‘other medical depts.’ We also folded together various hospital-wide clinical functions like nursing, imaging, and pathology into a category we called ‘hospital-wide’ for indicators about clinical activities that span the hospital. However we maintained a separate category rehabilitation services, for which France maintains 22 indicators, on the grounds that in French hospitals it is a discrete function In looking through the indicators it also became clear that there were many targeting various non-clinical ‘hospitality’ functions of a hospital, like catering and housekeeping. To represent them we created an additional category: ‘Non-clinical services’. We also added a category called ‘Management’ to refer to any indicators measuring the quality of administrative functions such as finance, human resources, ICT systems, hospital records. In total we had 20 discrete ways of classifying the particular hospital activities that a given indicator is assessing (Table C).

**Table C. Hospital activity categories used to classify quality indicators**

| A&E: Indicators targeting the quality of care in emergency departments |
| --- |
| Anaesthesia: Indicators assessing administration and control of pain relief |
| Cardiology: Indicators for the treatment of heart surgery and other cardio-vascular conditions, including, but excluding carotid surgery and stroke care |
| Gastroenterology: Indicators relating to treatments of the digestive system, including liver and pancreatic surgeries |
| Geriatrics: Indicators focused on the care of elderly and therefore potentially vulnerable patients |
| Intensive care (ICU): Indicators targeting care in intensive care units |
| Nephrology: Indicators for kidney and pancreas treatments, except for cancer |
| Neurology: Indicators for neurological conditions, including carotid surgery and stroke care |
| Obstetrics: Indicators relating to the treatment of both mother and baby in childbirth, including neonatal care |
| Oncology: Indicators for cancer care |
| Orthopaedics: Indicators concerned with treatment of musculoskeletal conditions |
| Outpatient care: Indicators focused on the quality of care delivered by hospitals to patients ‘at home’ or otherwise not admitted to wards |
| Paediatrics: Indicators focused on the care of children, but excluding neo-natal care |
| Psychiatry: Indicators for mental health services |
| Rehabilitation: Indicators for specific rehabilitation services to restore function and prepare patients for release from hospital |
| Respiratory medicine: Indicators for the care of community acquired pneumonia and other respiratory conditions, but excluding lung cancer |
| Other medical depts: indicators for clinical specialties that were unique to a particular indicator set and numbering less than 5 in total were lumped together under this category |
| Hospital-wide: Indicators for clinical activities that span multiple hospital departments or the entire hospital: e.g. nursing, infection control, pathology, imaging, etc |
| Non-clinical services: Indicators assessing the quality of patient-focused but non-clinical functions and services like catering, parking, visiting hours, and other ‘hospitality’ functions |
| Management: Indicators targeting hospital administration, finances and governance |

Applying this classification framework could challenging when a particular indicator targeted an activity at the intersection of two specialities. For indicators about neonatal care, we classified those pertaining to the birth as obstetrics and the rest as paediatrics. We classified indicators pertaining stroke care and carotid surgery as ‘neurology’ rather than ‘cardiology.’ For cancer, we defaulted to oncology, classifying the Dutch indicators about lung cancer (9.3 Lung carcinoma in [8]) under that category rather than ‘respiratory medicine’.

The complete set of indicators and our classification of them is available in the supplementary materials for this paper.

# REFERENCES

1 Donabedian A. The Quality of Care: How Can It Be Assessed? *JAMA* 1988;**260**:1743–8. doi:10.1001/jama.1988.03410120089033

2 CQC. Intelligent Monitoring: NHS Acute Hospitals Indicators and Methodology—May 2015. London: : Care Quality Commission 2015.

3 Institute of Medicine, Services B on HC, Delivery C on the NQR on HC. *Envisioning the National Health Care Quality Report*. National Academies Press 2001.

4 Kelley E, Hurst J. Health Care Quality Indicators Project. 2006. http://www.oecd-ilibrary.org/social-issues-migration-health/health-care-quality-indicators-project_440134737301 (accessed 1 Feb 2018).

5 WHO. Performance Assessment Tool for Quality Improvement in Hospitals. Copenhagen: : World Health Organization 2007. http://www.euro.who.int/__data/assets/pdf_file/0003/103728/E89742.pdf

6 Arah O, Westert G, Hurst J, *et al.* Conceptual framework for the OECD Health Care Quality Indicators Project. *Int J Qual Health Care* 2006;**18**:5–13. doi:10.1093/intqhc/mzl024

7 Ettelt S. The politics of evidence use in health policy making in Germany: The case of regulating hospital minimum volumes. *J Health Polit Policy Law* 2017;**42**:513–38. doi:10.1007/978-3-319-93467-9_6

8 IGZ. Basisset kwaliteitsindicatoren ziekenhuizen, 2016. Utrecht: : Inspectie voor de Gezondheidszorg 2015. https://www.igj.nl/binaries/igj/documenten/indicatorensets/2015/08/06/basisset-kwaliteitsindicatoren-ziekenhuizen-2016/IGZ+Basisset+kwaliteitsindicatoren+ziekenhuizen+2016_tcm294-367407.pdf
